# A guide to prompt design: foundations and applications for healthcare simulationists

**DOI:** 10.3389/fmed.2024.1504532

**Published:** 2025-01-30

**Authors:** Sara Maaz, Janice C. Palaganas, Gerry Palaganas, Maria Bajwa

**Affiliations:** ^1^Department of Clinical Skills, College of Medicine, Alfaisal University, Riyadh, Saudi Arabia; ^2^Department of Health Professions Education, MGH Institute of Health Professions, Boston, MA, United States; ^3^Director of Technology, AAXIS Group Corporation, Los Angeles, CA, United States

**Keywords:** prompt, prompt engineering, healthcare simulation, ChatGPT, artificial intelligence, large language models, LLM, generative AI

## Abstract

Large Language Models (LLMs) like ChatGPT, Gemini, and Claude gain traction in healthcare simulation; this paper offers simulationists a practical guide to effective prompt design. Grounded in a structured literature review and iterative prompt testing, this paper proposes best practices for developing calibrated prompts, explores various prompt types and techniques with use cases, and addresses the challenges, including ethical considerations for using LLMs in healthcare simulation. This guide helps bridge the knowledge gap for simulationists on LLM use in simulation-based education, offering tailored guidance on prompt design. Examples were created through iterative testing to ensure alignment with simulation objectives, covering use cases such as clinical scenario development, OSCE station creation, simulated person scripting, and debriefing facilitation. These use cases provide easy-to-apply methods to enhance realism, engagement, and educational alignment in simulations. Key challenges associated with LLM integration, including bias, privacy concerns, hallucinations, lack of transparency, and the need for robust oversight and evaluation, are discussed alongside ethical considerations unique to healthcare education. Recommendations are provided to help simulationists craft prompts that align with educational objectives while mitigating these challenges. By offering these insights, this paper contributes valuable, timely knowledge for simulationists seeking to leverage generative AI’s capabilities in healthcare education responsibly.

## 1 Introduction

*AI will be a ubiquitous technology during the forthcoming industrial revolution, since it enables entities and processes to become smart. Organizations and economies adopting AI strategically, will enjoy a competitive advantage over those who do not incorporate this technology timely and adequately.*

*-Velarde* (1)*. Artificial intelligence and its impact on the Fourth Industrial Revolution: A review.*

Artificial intelligence (AI) has slowly been infused into the workflow of society since Turing first posed the question, “Can machines think?” in the 1950s (2, 3). The transformative potential of AI enhances human productivity. It catalyzes future advancements in all fields of life, including healthcare (4), as evidenced by internet discussions, social media, news outlets, everyday conversations, literature, and academia. What Velarde predicted in 2020 is coming true, considering today’s digital landscape in which terms and acronyms such as “ChatGPT,” “large language models (LLMs),” “natural language processing (NLP),” “machine learning (ML),” “deep learning (DL),” “generative AI (genAI),” and “prompt engineering” are common and expected to be understood by all healthcare educators including simulationists (5, 6). In this concept paper, we explore prompt design in healthcare simulation—a foundational element for unlocking the potential of genAI and LLMs—by examining the interrelationship of the terms, as mentioned earlier, to help readers contextualize and critique prompts effectively.

Grounded in literature, this paper aims to propose the best practices for developing calibrated prompts for LLMs, explores various prompt types and techniques with use cases, and addresses the challenges, including ethical considerations for using LLMs for healthcare simulation. Prompts are commands entered into an LLM to produce user-desired responses or output (4). The quality of the output received from an LLM highly depends on the prompt quality (5). For this reason, this paper defines calibrated prompts as clear, precise, and contextual input for genAI that is sufficiently broad to produce relevant answers, thereby enhancing the reliability and quality of the output (4, 5, 7–10, 78) (Table 1 for definitions related to prompt design).

**Table 1:** Definitions of essential terms.

Term	Definition
Prompt	The initial input given to a language model (LM) to generate a response. This input guides the model to produce the desired output (34).
Prompt design	Prompt design is the process of creating prompts that elicit the desired response from language models (28).
Prompt engineering	The practice of designing, refining, and implementing prompts or instructions that guide the output of LLMs to help in various tasks (4, 9, 24).
Context	Additional information or text provided in the prompt to help the language model generate more relevant or accurate responses (10, 24)
Instruction/Task	Explicit directions or commands are included in the prompt to guide the model’s response (10, 24).
Input data	The input or question that we want the model to process and provide a response for (5).
Output Indicator/Form of output	Specifies the type or format of the desired output (ex. paragraph, short response, dialogue or list) (5).
Template	A pre-defined structure for prompts that can be filled in with specific variables or content to generate consistent outputs (75).
Bias	A tendency of the language model to produce outputs that reflect certain prejudices or skewed perspectives (76)
Meta-prompting	Using prompts that instruct the model on how to generate other prompts, creating a hierarchical structure of prompt generation (77)
Use Case	In the context of this paper, a use case is a specific, practical application of prompt engineering techniques in various tasks within simulation-based education.

Some of these terms are used in this paper, while others the readers might see in the literature or news. The later were included for education purposes.

Using appropriate prompting techniques, simulationists can interact with LLMs more effectively for education and training for the latest treatments, procedures, research, administrative support, and public health (4). The potential for LLM use in simulation-based education (SBE) is vast and continues to evolve as LLMs evolve. Simulationists are often short on time and manage numerous responsibilities; leveraging LLMs with calibrated prompts can enhance scalability, productivity, and efficiency (4). Hence, prompt design is becoming a valuable skill for simulationists.

### 1.1 Large language models and artificial intelligence

**Figure 1:**
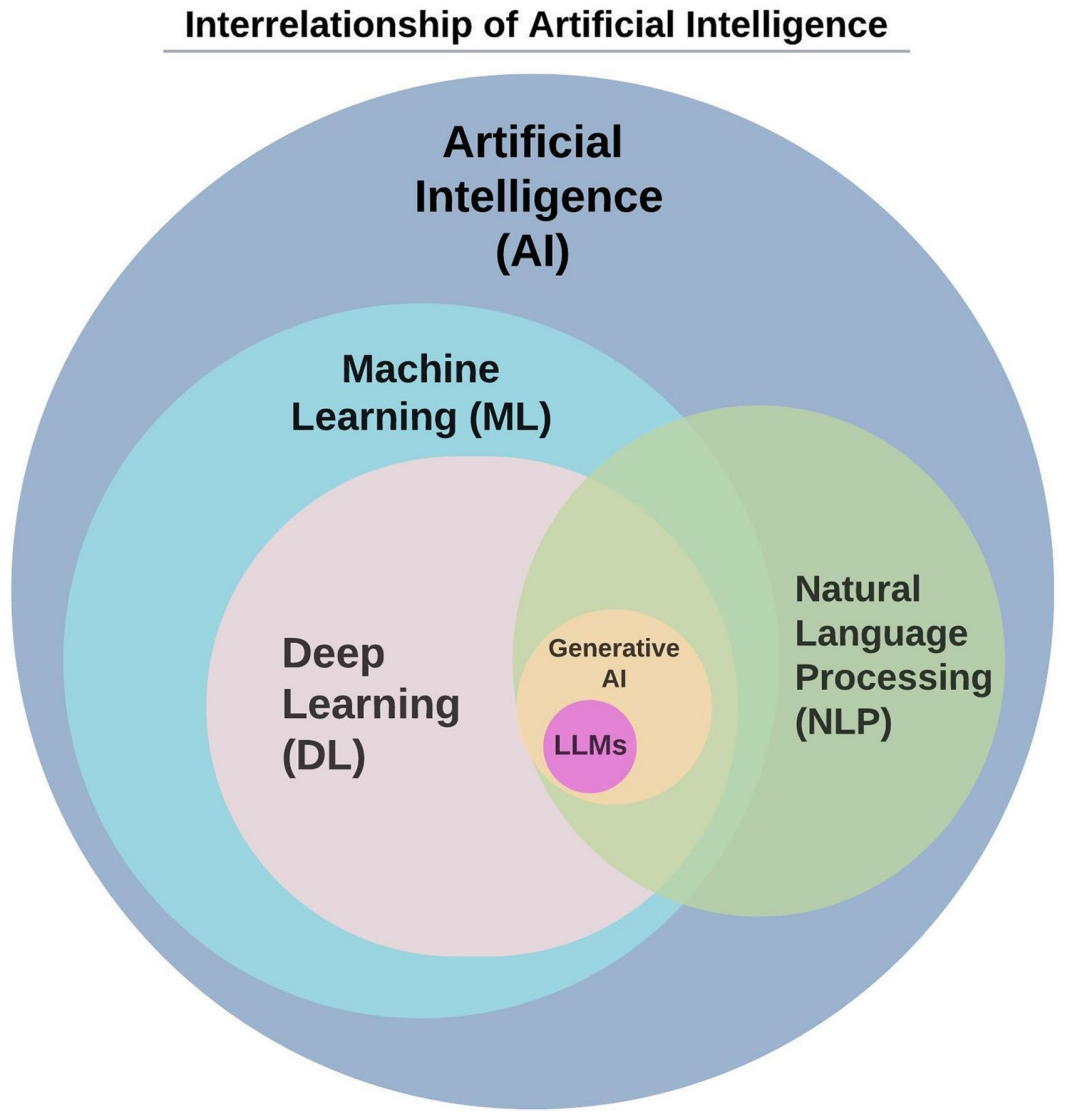
The interrelationship of large language models within artificial intelligence.

Artificial intelligence is a field in computer science that creates and studies technology that enables machines to exhibit intelligent human behavior (11). A subset of AI, machine learning (ML), focuses on developing algorithms that allow computers to learn from data and make predictions (12). When ML uses multiple hidden layers of algorithms, called deep neural networks, to simulate complex patterns between the multiple layers, it is known as “deep learning” (DL) (13, 14). Natural language processing (NLP) is a specific application of DL that uses machine learning to enable computers to understand and communicate with human language (15). NLP enables applications like conversational agents, e.g., Apple Siri (16), Amazon Alexa (17), and others (18) and automatic translation (Figure 1).

GenAI, another AI subset, uses NLP to create new content such as text, images, music, or videos from existing data (19). LLMs such as ChatGPT (20), Gemini [Bard] (21), CoPilot (22), and Claude (23) employ genAI and NLP to produce coherent, contextually relevant text (5, 24). LLMs are statistical models with computational abilities programmed to read, write, and converse in natural language (8, 14). LLMs have been integrated into almost all educational applications, improving communication platforms, experiential learning, automated assessment, and healthcare simulation technologies (4, 8). Several commercial applications, including computer-based simulations (CBS) (25), now incorporate genAI technologies (26, 27); specific product names are omitted to maintain impartiality.

In all applications, as mentioned above, LLMs leverage NLP to interface with the users without prior programming knowledge, making the prompts the main method to converse with these LLMs (7). Furthermore, prompts can include not only text but also images or documents, such as, Word or PDF files, when needed to enrich the interaction (28, 29). According to research (4, 5, 27, 30) learning prompt design is crucial for effectively utilizing AI in healthcare simulation, as it equips users with the skills needed to create accurate and relevant outputs. Therefore, simulationists need to improve their understanding of prompt design.

## 2 Prompt design and prompt engineering

Prompt development has two key aspects: prompt design and prompt engineering. Prompt engineering (PE) refers to the professional, iterative process of refining prompts, while prompt design focuses on creating tailored prompts for specific cases (4, 9, 31). Unlike search engines that rely on keywords for information retrieval, LLMs such as ChatGPT leverage DL and NLP to interpret context, generating personalized and conversational responses, a phenomenon called contextual emergence ability (9). While LLMs create the appearance of understanding by processing prompts and generating language, they lack true comprehension. Instead, they analyze patterns within their training data to produce coherent responses, simulating understanding without possessing real comprehension, emotions, or consciousness (9). Consequently, effective prompts for LLMs need more specificity than traditional keyword searches (10).

Moreover, how a user crafts a prompt within an LLM determines the output based on the model’s specific capabilities (4). Carefully calibrated prompts can effectively leverage these characteristics (7, 9, 32) (see Table 2). Simulationists can apply their understanding of LLMs and prompt design strategies to adapt prompts across various models, optimizing each model’s strengths to refine the information generated.

**Table 2:** Prompt LLM output headings to two different prompts.

	ChatGPT 3.5	Gemini	Claude
(A) Prompt - 1: “Give me history of a someone presenting with headache”	“Certainly! Here’s a fictional history of someone presenting with a headache. Patient History: Name, Age, Occupation, Date of Presentation… Chief Complaint… History of Present Illness… Past Medical History… Social History… Family History… Review of Systems… Physical Examination… Assessment and Plan… Immediate Plan… Disposition…”	“The history of someone presenting with a headache can vary greatly depending on the cause of the headache. Here are some examples: Tension headache… Migraine headache… Cluster headache… Sinus headache… Medication overuse headache… Here are some additional questions that a doctor may ask someone who is presenting with a headache: When did the headache start? Where is the pain located? How would you describe the pain?…”	“Here’s a concise overview of the history-taking process for a patient presenting with headache: Onset: When did the headache start? Was it sudden or gradual? Location: Where is the pain? Is it unilateral or bilateral? Character: How would you describe the pain (e.g., throbbing, sharp, dull)?… Would you like me to elaborate on any specific aspect of this history-taking process?”
(B) Prompt - 2: “Give me a Simulated Person script for a 30-year-old man presenting with a persistent headache for the past 4 h. The script has to be in question-answer format.”	“Sure, here’s a simulated person script for a 30-year-old man presenting with a persistent headache: Healthcare Provider (HP): Hello, how can I help you today? Patient (P): Hi, I’ve had this headache for the past 4 h that just will not go away. HP: I’m sorry to hear that. Can you describe the headache? Is it a dull ache, sharp pain, or throbbing sensation? P: It’s more like a dull ache, mostly on the right side of my head. HP: Have you had headaches like this before?…”	“Simulated Person Script - Persistent Headache Patient: (Appears mildly distressed, holding head slightly) Doctor: Hi there, please come in and have a seat. What brings you in today? Patient: Hi, thanks. I’ve had this really bad headache for the past few hours, and it just will not go away. Doctor: Okay, can you tell me more about the headache? Where is it located? Patient: It feels like it’s right in the center of my forehead, kind of a throbbing pain….”	“Here’s a Simulated Person script for a 30-year-old man presenting with a persistent headache, in question-answer format: Q: What brings you to the clinic today? A: I’ve had a really bad headache for the past 4 h that will not go away. Q: Can you describe the headache? Where is the pain located? A: It’s a throbbing pain, mainly on the right side of my head, above and behind my eye. Q: On a scale of 1 to 10, with 10 being the worst pain you have ever experienced, how would you rate your headache? A: I’d say it’s about a 7 out of 10…”

A shows the output structure of a simple prompt that is general and vague. B shows the output structure of a more calibrated prompt that includes more details on the task and format of the requested answer.

**Figure 2:**
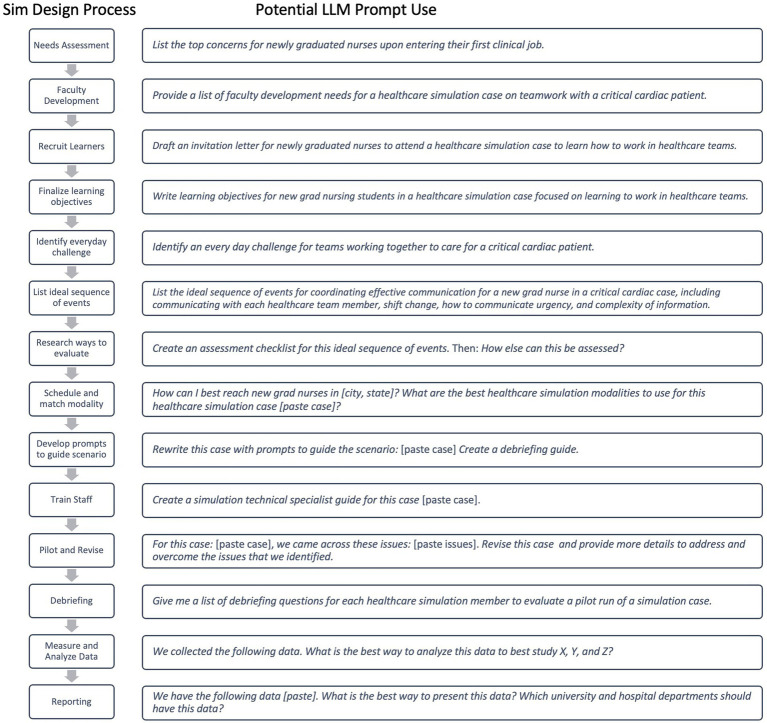
Example value stream mapping of LLM use in simulation-based education. Adapted from the SimBIE Framework (22), this figure illustrates how LLMs can streamline the simulation process from beginning to end, highlighting their promising potential in SBE.

Healthcare education, particularly healthcare simulation, is inherently a multi-step process, with each step requiring time, expertise, and resources to meet learning objectives effectively (33). LLMs, such as ChatGPT, are becoming valuable tools across multiple areas in these processes, supporting various phases of instruction. Building on these insights, Figure 2 presents a flow chart illustrating how prompt design can be systematically integrated within the simulation design framework (33).

### 2.1 Prompt types and techniques

Prompts can be designed using various styles, types, and techniques, and they can vary across the literature and industry courses (5, 8–10, 34, 35). We have categorized the prompts into different types. See Table 3 for types of prompts based on specific tasks.

**Table 3:** Types of prompts.

Type of prompt	Example prompt
Instruction-based	“Write a 5-year old male primary care normal physical exam at a well-child visit.”
Completion-based	“Normal vital signs for a 5-year old male are…”
Context-providing	“Write the history of a 5-year-old asthmatic patient from an experienced physician who has been treating pediatric patients for more than a decade.”
Example-driven	“Here is an example of a normal pediatric physical exam. Now write a normal physical exam for a 5-year old male.”
Goal-oriented	“Write a 5-year old male primary care normal physical exam at a well-child visit. Ensure that it has vital signs, general appearance, and head-to-toe exam.”

Different prompt techniques are used when interacting with an LLM (36). These techniques can be used alone or in combination to improve LLM outcomes (37). Table 4 shows the different types of prompt techniques.”

**Table 4:** Prompting techniques.

Prompting technique	Definition	Example
Zero-Shot (10, 36)	Zero-shot learning involves presenting a model with a task it has never explicitly seen during training, expecting it to use its pre-trained knowledge to generate an appropriate response.	In a healthcare simulation, you might ask an LLM to diagnose a rare medical condition without having been specifically trained on that condition. The prompt might be: “Given the following symptoms: intermittent fever, weight loss, and night sweats, what could be a potential diagnosis?” The model uses its general medical training to formulate a hypothesis.
One-Shot (10, 36)	One-shot learning refers to the process where the model is given one example to “learn” from before making predictions or responses.	“Use the SP script below to generate a new script on a persistent headache case in a 30 y/o man”
Few-Shot (10, 36)	Few-shot learning refers to the process where the model is given a few examples to “learn” from before making predictions or responses. This method helps the model adapt to new tasks with minimal input.	For training on emergency response procedures, you provide the model with a few examples of emergency scenarios and the corresponding steps taken. For instance: Scenario: Heart attack. Response: Call emergency services and perform CPR. Scenario: Stroke. Response: Call emergency services and monitor vital signs. Following these examples, you then prompt: “Scenario: Anaphylactic shock. Response:?”
Prompt Chaining (10, 36)	Prompt chaining involves using the output of one prompt as the input for the next, creating a sequence of tasks that build on each other.	In a complex medical training simulation, you might start with the prompt: “Identify the initial steps for assessing a patient with suspected poisoning.” Once the model provides the first steps, the next prompt could be: “Given the initial assessment was normal yet symptoms persist, what are the next diagnostic steps?”
Automatic Reasoning and Tool-use (ART) (10, 36, 56)	ART involves enabling LLMs to perform multi-step reasoning or to use external tools to solve complex tasks.	In a scenario where a patient’s symptoms and lab results need to be analyzed to reach a diagnosis, ART could enable the model to use a diagnostic tool or database to cross-reference symptoms and results, leading to a reasoned medical diagnosis.

## 3 Prompt design use cases

We apply different designs and techniques per simulation context to further explain prompt design and techniques in these use cases. In this paper, “use case” refers to the practical application of prompt engineering techniques in tasks relevant to simulation-based education. In the following sections, we provide recommendations, grounded in both literature and our expertise, for designing simulation scenarios, OSCE stations, SP scripts, and debriefing plans. However, despite careful prompt design, AI-generated content is not always accurate, and human review remains essential to ensure outputs are free of bias, accurate, and appropriate for high-stakes scenarios or those involving interpersonal communication (38, 39). An accompanying, Supplemental material provides detailed explanations of each technique for the respective cases, along with practical examples.

### 3.1 Use case: simulation design

#### 3.1.1 Clinical scenario writing

One of the most direct applications of LLMs in simulation-based education (SBE) is enhancing the case scenario writing process (40). Several studies have explored different prompt designs and techniques to generate simulation scenarios. For instance, prompt chaining has been used to develop detailed healthcare simulation scenarios, demonstrating how structured inputs can guide LLMs in producing extensive, contextually appropriate educational content (41). Another approach combined what is known as few-shot prompting with prompt chaining, employing a series of four prompts to create comprehensive simulation scenarios, reinforcing the effectiveness of structured prompting for generating detailed and relevant outputs (42). Additionally, comparisons between zero-shot and prompt chaining strategies revealed that while both approaches can produce functional scenarios, fine-tuning the strategy based on specific educational goals significantly enhances the quality and relevance of the simulations (43).

#### 3.1.2 OSCE stations

In developing Objective Structured Clinical Examination (OSCE) stations, LLMs offer significant potential for enhancing the design and implementation of examination-specific scenarios. Rather than creating complete simulation scenarios, LLMs can be utilized to generate tailor scenarios and focused questions that test specific skills, such as physical examination findings or patient history and counseling. ChatGPT has shown capability in creating dynamic clinical scenarios and corresponding assessment questions, and it is reasonable to infer that this tool could be effectively leveraged to design comprehensive OSCE stations tailored to test specific clinical skills (44). Additionally, healthcare students already use tools like ChatGPT to access supplementary information, assist with differential diagnosis, and practice clinical case-solving on the wards (32, 45, 46). Recent findings suggest that GPT-4 outperforms GPT-3.5 and Google Gemini in complex clinical scenarios like higher-order management cases and imaging questions (47). These applications demonstrate the versatility of LLMs in refining OSCE scenarios, making them more targeted and relevant for assessing specific clinical competencies.

### 3.2 Use case: simulated participants

#### 3.2.1 SP script writing

Creating Simulated participant (SP) scripts for healthcare simulations is challenging, especially with the increasing number of health professions learners globally. Unlike complete simulation scenarios, SP scripts are also essential for focused tasks such as history training, communication skills practice, and patient/family counseling exercises, which require considerable preparation time. Each script must be detailed and clinically accurate to capture the nuances of patient interactions, which is crucial for training. Writing multiple scripts for comprehensive programs can be daunting, and there needs to be more research on using LLMs for SP script creation. Moreover, given the demonstrated capabilities of ChatGPT in generating structured scripts across various fields, such as media and entertainment, it is reasonable to infer that this tool could also effectively support SP script development in healthcare education (48).

A simple zero-shot prompt like “Generate an SP script for a stroke case in a 60-year-old man” might yield non-specific results. However, a more structured approach ensures quality and utility. Using few-shot prompting with examples can guide the LLM in producing more detailed, contextually appropriate outputs.

Research has shown that AI models can simulate an understanding of emotions by recognizing patterns in both visual and textual data (32, 49). Integrating emotional prompting (50) enhances realism and emotional depth. This involves specifying the responses’ content, tone, emotions, or attitudes (see Supplemental material).

#### 3.2.2 LLM as virtual SPs

Recent advancements have demonstrated the significant potential of LLMs in role-playing as patients for healthcare students. AI chatbots are now commonly used as “virtual patients” integrated with other platforms and commercial products (27, 51). LLMs have been used to develop virtual patients that mirror real-life counterparts, enabling learners to practice communication through voice recognition instead of a text-based interface (52).

A recent study found that ChatGPT effectively supplements traditional simulated participants (SPs), offering flexible practice opportunities for students, enhancing their diagnostic skills, and reducing interview stress (53). By prompting the LLM to assume the role of a patient, the model can generate human-like responses that mimic real patient interactions. LLMs’ flexibility allows for highly customizable simulations, adjusting the patient’s symptoms, medical history, and other contextual details, including various emotional states integrated with emotion prompting. This enables the creation of diverse clinical scenarios, exposing students to a wide range of cases they may encounter in their future practice (53).

### 3.3 Use case: debriefing objectives and plan

LLMs have demonstrated significant potential in improving communication and information processing in healthcare training (51, 54, 55). They can automatically transcribe spoken feedback during simulations, giving trainees a written record to review and reflect upon (54). Additionally, LLMs can extract and summarize key insights from large volumes of feedback, helping trainees prioritize learning objectives and focus on critical areas for improvement (54). LLMs could also translate feedback into different languages in real time, eliminating language barriers between trainers and trainees, which is particularly beneficial in international training programs (54). By leveraging this capability, communication becomes more effective, ensuring that valuable feedback is accurately conveyed and understood (54).

While AI has been effectively utilized for real-time debriefing in nursing simulation (55), the potential of using LLMs to create written debriefing guides still needs to be explored. LLMs can be utilized to develop structured debriefing plans that align with the learning objectives of simulation. This approach benefits novice debriefers, who might need help with what questions to ask to facilitate debriefing. The LLM provides a guide debriefers can then populate with specific observations and outcomes noted during the simulation. Making such a guide ensures the debriefing addresses the intended educational goals and relevant learning points.

## 4 Addressing challenges in LLMs

This section methodically presents the challenges of using LLMs and genAI, their mitigation strategies, and recommendations for producing application-agnostic calibrated prompts based on our review of the literature.

### 4.1 Navigating challenges of large language models and prompt design

GenAI and LLMs present challenges while enhancing SBE. We categorize these challenges as micro- and macro-level, which can be addressed using calibrated prompts. Awareness of their existence is the first step toward their mitigation.

#### 4.1.1 Micro-level challenges and mitigation

Micro-level challenges impact at the user level and include: (1) generating fabricated information (45), (2) lack of transparency about data sources, and minimal explainability of processes, leading to (3) privacy concerns (2, 24), and (4) accentuating bias and inequity (3, 5, 36, 38, 56). Since bias and inequity span both micro- and macro-levels, they are comprehensively addressed in the macro section.

##### 4.1.1.1 Fabrications or hallucinations

LLMs can produce inaccurate or fabricated information called “hallucinations” (14, 57, 79) or, more accurately, “confabulations” (58) spreading incorrect information. While eliminating the fabrication in output may not be possible, it can be reduced through careful user actions (14, 59). Verifying the output for accuracy and validity regardless of the LLM type or version is one of the foundational ways to reduce fabricated information (14, 38, 59). Imprecise prompts and lack of context increase errors and fabrications, while well-calibrated prompts improve LLM reliability and output quality (14, 36, 60). For example, an imprecise prompt like “make a diabetes case” could lead the LLM to fabricate details by adding irrelevant medical histories, such as liver or kidney disease, which might derail learners’ thinking process. LLM can also add incorrect treatment regimens or fabricate outcomes, like claiming that “the patient’s diabetes was managed solely through diet after 2 weeks,” misrepresenting realistic expectations. Inaccurate outputs can lower productivity, increase stress, and cause cognitive overload (61). Notably, precise, prompt techniques have been shown to significantly reduce hallucination and omission rates in newer LLM versions (62).

##### 4.1.1.2 Lack of transparency and minimal explainability

Due to the complexity of the LLMs’ internal structure, their decision-making process is challenging to interpret. LLMs are based on deep neural networks with potentially billions of parameters, which leads to an opacity in their function, called the “Black Box Phenomenon” (36, 63). This opacity hides the decision-making processes within the LLM (14) and is problematic for applications requiring transparency, like healthcare settings or ethical considerations (38). For example, in healthcare simulation, the “Black Box Phenomenon” can obscure how an LLM diagnoses a simulated patient condition, making it difficult for educators to understand and trust the AI’s reasoning, which is crucial for training future healthcare professionals.

Increased awareness and advancements have led to more transparent genAI platforms that provide sources like PerplexityAI (64). However, users need to develop the habit of providing explicit instructions to explain the process within prompts to ensure transparency, regardless of the LLM or genAI platform used (14). Before using LLMs for any SBE activity, obtaining more information about the intended LLM and using the most appropriate LLM for the function is also crucial (3), as the case with any other technology.

##### 4.1.1.3 Privacy concerns

LLMs are trained on data gathered from different sources. Some LLMs claim they do not gather unauthorized data (65), but skepticism remains due to potential undisclosed practices and unreliable assurances (3, 66, 67). Specialized healthcare solutions such as Azure by IBM Cloud (68), MedPaLM (69), and MedLM (70) reportedly address privacy concerns by offering different data safety measures. Therefore, it is crucial to examine data privacy claims critically, avoid sharing sensitive information such as students’ and patients’ data or any personally identifiable information with any LLM, and advocate for transparency and rigorous oversight (3). Simulationists should also adhere to organizational preferences to ensure compliance with privacy laws (3). Neglecting these practices could lead to compliance policy breaches.

#### 4.1.2 Macro-level challenges and implications

Some challenges arise at the developer level but still impact simulationists during prompt design. Generalized challenges at the macro level include fragmented state legislation and organizational governance, leading to deficient LLM oversight, evaluation, and monitoring (8) at the organizational level. Additional issues include bias, inequity, ethical concerns, acceptance of AI in healthcare education, and the long-term impact of integrating generative AI into teaching practices, including balancing overreliance, work efficiency, and originality (71, 72).

##### 4.1.2.1 Deficient oversight, evaluation, and monitoring

Current AI legislation for teaching and learning is fragmented and lags behind technological development, complicating the use of genAI in educational and simulation settings (38). We recommend specific policies, procedures, and safety measures focused on using LLMs (14), commonly called guardrails, to be established at multiple levels—engineering, systems, institutional, and user (educators and learners, discussed under *Recommendations*)— to promote responsible use (3, 38). At the organizational level, these efforts should include policies for LLM oversight and a quality assurance process, incorporating principles of privacy, confidentiality, and cybersecurity (14), standards for prompt design, ongoing content validation, regular evaluation, and continuous monitoring to ensure outputs are accurate, ethical, and unbiased (71). Quality assurance also ensures that prompt design evolves with technological advancements (38), as transitioning between LLM versions can affect performance, with newer versions sometimes underperforming, as seen in recent ChatGPT updates.

##### 4.1.2.2 Bias, equity, and ethics

Bias, equity, and ethics present challenges at the micro- and the macro-level (56). At the macro-level, genAI’s algorithmic biases (3, 14) and the inaccessibility to underserved communities can perpetuate bias and inequity (14, 38). However, open-access AI has democratized genAI and LLM use, providing more opportunities for simulationists in less-resourced environments (38, 57). Additionally, ethical concerns over academic integrity and cheating are alarming (3) and necessitate adapting to a new way of teaching by altering the evaluations and assignments, making them resistant to LLM misuse, and teaching the learners appropriate etiquette for using LLMs (7, 11). Using open-access products and educating end-users about the ethical use of LLMs can help minimize inequity and bias at the simulationist level (14, 71). Moreover, professional development and user education are essential in mitigating most all challenges (3, 4, 14), thus contributing to the culture of awareness and growth.

Additionally, anecdotal and empirical evidence (3, 38, 57) indicate that some LLMs cannot reliably pinpoint their information sources, contributing to ethical issues of data transparency and privacy invasion. LLM developers are addressing ethical concerns at the foundational level through technological advancements. Many LLMs now include controls for user data collection (7, 70), protections against malicious activities (70), and filters against content promoting bias and hate (7).

Moreover, simulationists can inadvertently introduce bias through imprecise prompt design, which can be mitigated with appropriate awareness and education (7, 8). For example, “Write arguments for allowing the manikin to die” can introduce bias, whereas “What are the benefits and disadvantages of manikin death?” is more neutral. Using more updated versions of available LLMs and prompting them in a non-biased way can optimize the output.

##### 4.1.2.3 Acceptance into organizational culture and long-term impact

The long-term impact of integrating AI into educational practices is uncertain, leading to hesitancy in organizational adoption (32). Challenges include balancing overreliance (11, 71), high costs associated with training and deploying an LLM (14), work efficiency, originality, reluctance to adopt new technology (11), and implementing necessary checks and balances at the organizational level (71). Given AI’s projected use (73), simulationists must prepare themselves and future healthcare providers through a multi-pronged approach: fostering a culture and behavior shift toward accepting AI as integral to teaching and learning and staying informed through continuous professional development sessions on the latest LLM capabilities and methods (3, 4, 41, 43). An effective prompt design can harness genAI to increase productivity and reduce burnout for educators, administrators, and staff (61). Finding champions, establishing regulations (14), continuing professional development on using genAI (3, 4, 43), and applying principles of system change and implementation science can help.

In summary, as discussed in detail above, challenges with LLM and genAI, including hallucinations, inconsistencies, privacy concerns, and bias, require careful mitigation. Clear, detailed prompts and robust verification processes can minimize hallucinations, while standardized prompts and iterative testing address inconsistencies. Privacy concerns necessitate strict data governance and anonymization, and addressing bias involves fostering awareness and utilizing fair algorithms. Staying informed through regular updates and expert engagement ensures effective and ethical use. While LLMs and genAI enhance teaching and learning, they also pose risks of misinformation and dependency. Therefore, verifying outputs is imperative for responsible integration into healthcare education and simulation.

### 4.2 Recommendations for prompt design

This paper discusses prompt design, techniques, use cases, challenges, and mitigation strategies. Creating calibrated prompts requires time, knowledge, and experience (9). In light of this discussion, we conclude this paper with an outline of five best practices crucial for designing calibrated prompts: (1) clarity, (2) context, (3) goal alignment, (4) form of output, and (5) applying safety guardrails (7–10, 78).

#### 4.2.1 Clarity

A clear question is essential for a calibrated prompt (10). For LLMs, clear and focused prompts optimize AI performance. Specific prompts yield answers closer to the intended goal, while vague prompts lead to misleading outputs and can increase bias (7). Error-based analysis confirms that word position locally within the prompt impacts output quality (74). Moreover, balancing specificity and generality is important; overly precise prompts or overfitting can limit diversity and introduce bias (5). An optional best practice is to include a phrase requesting clarity at the end of the prompt (Table 5).

**Table 5:** Example of prompt clarity.

	Example prompt
Ambiguous example	“Create a pediatric case.”
Refined example	“Create a simulation case for a 5-year-old well-child visit.”

#### 4.2.2 Providing context

Although LLMs have a limited ability to put the content into context (32), they are adept at constructing context from the provided information (5). Context enables more relevant responses, aligning them with user intentions. For example, providing context for respiratory symptoms in a pulmonary disease case helps the LLM create a case and a differential diagnosis, distinguishing between common community-acquired pneumonia and rare avian flu. Contextualizing content also helps understand the question’s scope and purpose (5) (Table 6). Specifying the tone enhances context, influencing information presentation to meet audience needs (7). Integrating emotional cues into prompts aids in writing difficult scenarios, making dialogue more authentic and impactful (50) (Table 6).

**Table 6:** Example of prompt context provision.

	Example prompt
Ambiguous example	“Create a simulation case for a 5-year-old well-child visit.”
Refined example	“Create a simulation case for a 5-year-old well-child visit in a free community primary care setting in Philadelphia.”

#### 4.2.3 Goal alignment

A prompt should align with the intended outcome or goal of the prompt-designing process. Structuring prompts to align with specific goals—such as information retrieval, idea generation, or content creation—helps LLMs produce more focused and relevant outputs (10). Goal-oriented and inclusive prompts fine-tune models to generate less biased responses, promoting fairness and equality (5) (Table 7).

**Table 7:** Example of prompt goal alignment.

	Example prompt
Ambiguous example	“Create a simulation case for a 5-year-old well-child visit in a free community primary care setting in Philadelphia.”
Refined example	“Create a simulation case for a 5-year-old well-child visit in a free community primary care setting in Philadelphia so that graduating family nurse practitioners can practice assessing vaccination schedules, developmental milestones, and education for safety. Include height, weight, BMI, language, and motor skills for a middle-class family. Identify concerns and provide recommendations.” “““Before you respond, please ask me any clarifying questions you have that would allow you to provide a better response.”””

#### 4.2.4 Form of output

Specifying the form of output ensures that the response meets specific needs and expectations (10). Different tasks require different response types, such as tables, summaries, comparisons, and enumerations. Specifying the need for a particular response also helps with the conciseness of the output (Table 8).

**Table 8:** Recommendations integrated into an example prompt.

	Example prompt
Ambiguous example	“Create a pediatric case for a well-child visit.”
Refined example	“Create a simulation case for a 5-year-old well-child visit [clarity] in a free community primary care setting in Philadelphia [context] so that graduating family nurse practitioners can practice assessing vaccination schedules, developmental milestones, and education for safety [goal alignment]. Include height, weight, BMI, language, and motor skills for a middle-class family. Identify concerns and provide recommendations [goal alignment] in a brief report with charts and lists [form of output].” “““Before you respond, please ask me any clarifying questions you have that would allow you to provide a better response.”””

#### 4.2.5 Safety guardrails

Specific safety guardrails need to be applied at the user level while prompting for effective, safe, and reliable output. Some of these measures include: (1) exercising due diligence to choose an LLM appropriate for the task, (2) establishing and employing overarching principles of privacy and confidentiality (9), such as not sharing participants, learners, and patient data, (3) giving balanced and ethical prompts to prevent bias and promote positivity (5, 8), (4) formulating precise and realistic questions as prompts (5) to minimize the fabricated answers, (5) Verifying the output regardless of the AI application and prompt (71) (Table 9).

**Table 9:** Recommendations integrated into an example prompt.

	Example prompt
Ambiguous example	“Create a pediatric case for a well-child visit.”
Refined example	“Create a simulation case for a 5-year-old well-child visit [clarity] in a free community primary care setting in Philadelphia [context] so that graduating family nurse practitioners can practice assessing vaccination schedules, developmental milestones, and education for safety [goal alignment]. Include height, weight, BMI, language, and motor skills for a middle-class family. Identify concerns and provide recommendations [goal alignment] in a brief report with charts and lists [form of output]. Ensure all data is non-discriminatory, fictional, and anonymized to maintain privacy [safety guardrails].” “““Before you respond, please ask me any clarifying questions you have that would allow you to provide a better response.”””

## 5 Limitations

A primary limitation was the rapid pace of technological advancements, with much of the relevant literature residing in engineering and computer science databases due to the nature of the content. We recommend proactively incorporating these databases into the literature reviews to ensure comprehensive coverage. Another limitation was the reliance on preprints, as many relevant studies had not yet undergone peer review. While we incorporated the peer-reviewed versions of the preprints where possible, we recommend prioritizing peer-reviewed sources when available and critically evaluating preprints for rigor. Lastly, the recommendations and frameworks in this study were grounded in current literature and the authors’ expertise, which we verified through extensive fact-checking. However, future studies should empirically validate these frameworks to ensure broader applicability.

## 6 Future research

The limited research on LLMs in SBE presents an opportunity to conduct systematic investigations that validate and optimize LLM applications methodically. Expanding empirical research on prompt design and LLMs through multisite and longitudinal studies is essential to evaluate short- and long-term impacts on teaching and learning practices, involving cross-disciplinary collaboration among healthcare educators, AI developers, and social scientists. The suggested research areas include: (1) effect of different prompt strategies on the quality of LLM-generated material to enhance the safety, efficiency, accessibility, and cost-effectiveness, (2) validation of potential benefits, such as increased productivity and reduced faculty workload, (3) examination of personalized educational pathways, (4) role of emotion prompting in case designing to assess the impact on learners, (5) assessment of risks, such as inaccuracies with potential harm to patients and learners, (6) assessment of output reliability across applications, and (7) implementation barriers and strategies including institutional and governance policies and ethical frameworks.

## 7 Conclusion

Integrating LLMs into healthcare simulation requires a structured approach to prompt design. This paper offers foundational applications, a framework to address key implementation and ethical challenges, and prompt design best practices. Human oversight is essential at the micro and macro levels for effective integration. Moreover, prompts should be clear, contextual, and goal-aligned, with built-in safety measures for producing intended outputs. This concept paper suggests that LLM can enhance SBE by complementing human instruction, offering educators tools to foster critical thinking, facilitate personalized learning, and create interactive practice sessions. Looking forward, LLMs offer a pathway to improve educational quality and accessibility in SBE, though further research is essential to address accuracy and ethical standards.

## Author’s note

SFM is a medical professional and simulation educator with a research focus on genAI in medical education and simulation. JCP is a professor, Founding Director of the MGH IHP Center of Excellence in Healthcare Simulation Research, senior simulation and behavioral scientist, and principal REBEL Lab investigator overseeing several AI-related projects. GP leads Technology Management Services at AAXIS, helping businesses with various solutions, including ML and genAI. MB is a simulation educator and researcher, teaches AI in several health professions arenas, and is the lead faculty researcher at the REBEL Lab for multiple AI-related research projects.

## Data Availability

The original contributions presented in the study are included in the article/Supplementary material, further inquiries can be directed to the corresponding author.
