# Supplementary material for: A guide to prompt design: foundations and applications for healthcare simulationists
Source: Front Med (Lausanne). 2025 Jan 30;11:1504532. doi: 10.3389/fmed.2024.1504532 (PMC11841430; doi:10.3389/fmed.2024.1504532)
Supplement: Supplementary file 1 [file Data_Sheet_1.PDF]

A Guide to Prompt Design: Foundations and Applications for Healthcare Simulationists

Sara Maaz,[1,2] Janice Palaganas[1] Gerry Palaganas[3] Maria Bajwa[1]

Supplemental Material

This supplemental material provides a structured approach to prompt design in healthcare simulation, outlining practical techniques for creating scenarios, OSCE stations, SP scripts and debriefing plans using Large Language Models (LLMs) like ChatGPT's free version, GPT-3.5. Each table presents key prompting strategies, complete with explanations and examples.

Use Case: Simulation Scenario Writing

Prompting Technique	Explanation	Example
**One-shot/Few-Shot Prompting**	Begin by providing one or more examples to establish foundational context for the LLM, setting an initial framework for the scenario development process.	"You are an expert in simulation case writing. Review the simulation case below and understand its components and outline." Then, provide one or more high-quality scenario/s in the same prompt to serve as reference.
**ART Prompting**	Introduce specific evaluation tools, such as the Simulation Scenario Evaluation Tool (SSET)*, early in the prompting sequence to establish evaluative benchmarks and ensure alignment with educational goals.	"Review the attached/below evaluation tool elements and items, and understand them." This prompt ensures the LLM is aware of the criteria for scenario quality and educational value before developing the case further.

**Prompt Chaining**	Sequentially build upon each element of the scenario using a series of prompts, with each prompt adding detail or expanding on specific aspects according to the SSET guidelines and scenario requirements.	- "I need a simulation case on Myocardial infarction. The case has to fulfill all elements from the evaluation tool above. Start with the objectives and target learners."<br>- "Continue with the scenario, patient states, and critical actions."<br>- "Create a complete list of materials and equipment for the simulation."<br>- "Create a debriefing plan, objectives, and supporting evidence."
**Hybrid Prompting Strategy**	Combines few-shot examples, ART (Assessment-Referenced Technique), and prompt chaining for a structured, comprehensive approach to simulation case design that maintains alignment with evaluation tools and educational goals throughout the process.	This approach results in simulation scenarios that are both realistic and tailored to meet specific training objectives, effectively leveraging GPT-4's capabilities to develop robust medical training content that is educationally valuable.

* Hernandez J, Frallicciardi A, Nadir NA, Gothard MD, Ahmed RA. Development of a simulation scenario evaluation tool (SSET): Modified Delphi study. BMJ Simul Technol Enhanc Learn. 2020;6(6):344–350.

A Guide to Prompt Design: Foundations and Applications for Healthcare Simulationists

Sara Maaz,[1,2] Janice Palaganas[1] Gerry Palaganas[3] Maria Bajwa[1]

Use Case: OSCE

Prompting Technique	Explanation	Example Prompt
**Give the LLM Context**	Provide foundational information to help the LLM understand the requirements of the OSCE station, including the level of the learner, the focus of the assessment (objectives), and specific case details.	"You are a clinician tasked with writing an OSCE station third-year medical students on gastric ulcer. The scenario should focus on history-taking, physical examination, and initial counseling. I will provide further details to structure the station effectively."
**One-Shot/Few-Shot**	Supply one or more similar OSCE station/s as example/s, so the LLM can replicate structure, flow, and level of detail. The example should cover all key components, such as patient complaint, history, exam findings, and tasks for the student.	"Review the attached OSCE station and use the same structure to build the OSCE statio based on my coming prompts"
**Prompt Chaining**	Use a series of prompts to iteratively build and refine the OSCE station components. Each prompt develops specific aspects, ensuring detail and alignment with the learning objectives.	- "Create a patient scenario based on the provided objectives, including the patient's chief complaint and background."<br>- "Develop patient information, such as age, occupation, relevant history,

A Guide to Prompt Design: Foundations and Applications for Healthcare Simulationists

Sara Maaz,[1,2] Janice Palaganas[1] Gerry Palaganas[3] Maria Bajwa[1]

	While a single prompt might give a general overview, prompt chaining provides a structured, thorough output that meets the high standards needed for OSCE design. For tasks requiring layered details and precision, the multi-step approach aligns best with the LLM's strengths	and social habits."<br>- "Write instructions for the student, specifying tasks like taking history and counseling."<br>- "Draft a standardized patient script, including suggested responses and non-verbal cues."<br>- "List the key actions expected of the student, covering essential history questions, examination steps, and counseling points."<br>- "Describe expected physical findings and diagnostic information available to the student, such as lab results or imaging."<br>- "Set an appropriate time limit based on tasks like history-taking and examination."<br>- "List materials and equipment needed for this station"

A Guide to Prompt Design: Foundations and Applications for Healthcare Simulationists

Sara Maaz,[1,2] Janice Palaganas[1] Gerry Palaganas[3] Maria Bajwa[1]

Use case: Standerdized Patient Script Writing

Technique	Explanation	Example Prompt
**One-Shot/Few-Shot**	Provide a complete example SP script or several examples to establish a clear structure, tone, and level of detail for the LLM. This guides the LLM in producing contextually appropriate responses and realistic patient interactions.	"Review the provided SP script for a 60-year-old male with hypertension presenting with chest pain. Use this as a model to create an SP script for a 55-year-old female with similar symptoms, maintaining similar detail, structure, and tone. Include patient history, initial dialogue, non-verbal cues, and patient-initiated questions."
**Prompt Chaining**	Use a sequence of prompts to build and refine each element of the SP script, ensuring depth and accuracy. This approach develops the patient's profile, responses, emotions, and interaction cues in a structured way.	**- Patient Profile and Scenario Setup**: "Start with an introductory paragraph that describes the patient's background, main complaint, and basic demographic details.<br><br>**-Patient Responses**: "Create a standardized patient (SP) script for a clinical simulation in a structured question-and-answer format. The SP should respond in the first person, using direct dialogue for each response. Organize the script as a series

A Guide to Prompt Design: Foundations and Applications for Healthcare Simulationists

Sara Maaz,[1,2] Janice Palaganas[1] Gerry Palaganas[3] Maria Bajwa[1]

		of questions from the clinician followed by the SP's responses.The patient should be hesitant to discuss alcohol use but provides details after questioning."<br>- **Non-Verbal Cues:** "Include non-verbal cues in brackets, such as '[The patient fidgets and avoids eye contact when discussing family history].'"<br>- **Emotional Progression:** "Start with a neutral tone and gradually introduce signs of anxiety when discussing potential diagnoses or treatments."<br>- **Patient-Initiated Questions**: "Add patient questions, such as, 'Do you think this will require long-term medication?' and 'What lifestyle changes should I consider?'"

A Guide to Prompt Design: Foundations and Applications for Healthcare Simulationists

Sara Maaz,[1,2] Janice Palaganas[1] Gerry Palaganas[3] Maria Bajwa[1]

Emotion Prompting Examples:

Technique	Example
**Direct Emotional Instructions:**	Specify the emotion directly in the prompt. For example, "Generate an SP script where the patient is initially anxious about their diagnosis but becomes reassured after understanding the treatment plan."
**Contextual Emotions:**	Embed the desired emotional tone within the context of the scenario. For example, "Write a dialogue for a patient who has just received a serious diagnosis and is struggling to cope with fear and uncertainty."
**Emotional Descriptors:**	Use adjectives and adverbs in your prompts to guide the emotional tone of the responses. For example, "Create a patient interaction where the patient speaks softly and appears visibly distressed about their symptoms."
**Prompt Chaining for Emotional Depth:**	Use prompt chaining to develop the emotional arc of the patient throughout the scenario. Start with a prompt setting a neutral tone, and through a series of prompts, guide the LLM to shift the tone towards the desired emotional state.

frontiers

A Guide to Prompt Design: Foundations and Applications for Healthcare Simulationists

Sara Maaz,[1,2] Janice Palaganas[1] Gerry Palaganas[3] Maria Bajwa[1]

Use Case: Debriefing Guide

Prompt Technique	Explanation	Example Prompt
**One/Few-Shot**	This technique involves providing LLMs with one or more examples of debriefing guides from other simulations. The LLM learns the structure, key elements, and types of reflective questions that facilitate learning.	- "You are an expert in simulation-based education and debriefing. Review the attached debriefing guide from a previous simulation, noting its structure and reflection-based questions."<br>- "Here is a detailed simulation case involving a pediatric emergency. Review the case and objectives, and highlight any areas to prioritize in the debriefing based on the learning goals."<br>- "Using the learned structure and case details, generate a debriefing plan that includes reflection-focused questions and supports the learning objectives."

A Guide to Prompt Design: Foundations and Applications for Healthcare Simulationists

Sara Maaz,[1,2] Janice Palaganas[1] Gerry Palaganas[3] Maria Bajwa[1]

**Prompt Chaining**	This technique can progressively develop debriefing content, starting with the learned structure. The LLM can be prompted to expand specific sections, ensuring each part addresses the simulation's objectives and critical reflection areas.	- "Expand on the section discussing communication errors during the simulation, providing reflection questions and specific suggestions for improvement."<br>- "Incorporate prompts for trainees to reflect on their clinical reasoning in the scenario, focusing on decision-making steps and possible improvements."
